# An Updated Meta-Analysis of the Association between Tumor Necrosis Factor-α -308G/A Polymorphism and Obstructive Sleep Apnea-Hypopnea Syndrome

**DOI:** 10.1371/journal.pone.0106270

**Published:** 2014-09-15

**Authors:** Anyuan Zhong, Xiaolu Xiong, Huajun Xu, Minhua Shi

**Affiliations:** 1 Department of Respiratory Diseases, the Second Affiliated Hospital of Soochow University, Suzhou, China; 2 Department of Endocrinology, the Affiliated Drum Tower Hospital, Nanjing Medical University, Nanjing, China; 3 Department of Otolaryngology, Shanghai Jiao Tong University Affiliated Sixth People’s Hospital, Otolaryngology Institute of Shanghai Jiao Tong University, Shanghai, China; University of Thessaly School of Medicine, Greece

## Abstract

**Background:**

Several studies have reported that the tumor necrosis factor-α (TNF-α) -308G/A polymorphism is associated with susceptibility to obstructive sleep apnea-hypopnea syndrome (OSAHS). However, these results are controversial and conflicting.

**Objective:**

To evaluate the association between TNF-α-308G/A and OSAHS risk by meta-analysis.

**Methods:**

Electronic databases, including PubMed, Embase, China National Knowledge Infrastructure (CNKI), Wanfang, and Weipu, were searched to identify relevant studies. Data were extracted from the included studies. A model-free approach using odds ratio (OR), generalized odds ratio (ORG) and 95% confidence interval (CI) of the allele contrast to assess the association between the -308G/A polymorphism and OSAHS risk. Cumulative and recursive cumulative meta-analyses (CMA) were also carried out to investigate the trend and stability of effect sizes as evidence accumulated.

**Results:**

Seven studies including 1369 OSAHS patients and 1064 controls were identified in this meta-analysis. Significant associations were derived from the variants of the allele contrast [(OR, 1.78; 95% CI, 1.45–2.18) or (ORG, 2.01; 95% CI, 1.27–3.19). CMA showed a trend of an association. Recursive CMA indicated that more evidence is needed to conclude on the status of significance. No significant publication bias was found.

**Conclusions:**

Our meta-analysis suggested that the TNF-α-308G/A polymorphism contribute to the risk of OSAHS. Further studies with larger sample should be performed to confirm our findings.

## Introduction

Obstructive sleep apnea-hypopnea syndrome (OSAHS) becomes a major public health problem all over the world recently. OSAHS is a respiratory disorder characterized by upper airway obstruction during sleep, breathing pauses with oxygen desaturation, and arousal from sleep. The prevalence of OSAHS is 2% in females and 4% in males of middle-aged groups [1]. Studies show that OSAHS is an independent risk factor for development of cardiovascular, thrombotic and metabolic events [2]–[3]. Inflammation plays a central role in this pathophysiological process. Tumor necrosis factor (TNF)-α is a potential pro-inflammatory cytokine elevated on OSAHS patients and exerted multiple physiologic effects on sleep fragmentation [4], [5]. Also, chronic intermittent hypoxia (CIH) impairs endothelium-dependent vasodilator mechanism by stimulating NF-κB-mediated TNF-α generation [6]. Apparently, understanding the interaction between OSAHS and TNF-α is essential for developing therapeutic strategies.

Epidemiological studies confirmed that OSAHS is a chronic complex disease with strong genetic components [7], [8]. Among OSAHS-related candidate genes, TNF-α has been studied extensively. TNF-α gene is located on chromosome 6 (6p21), with several polymorphisms [e.g., -238 G/A (rs361525), -308 G/A (rs1800629), -857G/A (rs1799724) and -1031 T/C (rs1799964)] in the promoter region has been identified [9]. Among all the gene loci, -308 G/A was mostly studied [10]–[16].

However, the association between TNF-α-308 G/A polymorphism and OSAHS susceptibility still remained unclear, with inconsistent results from different studies. Also, each study has limitations in small sample sizes and low statistical power. In order to shed some light on this controversy, a meticulous meta-analysis, including a cumulative and a recursive cumulative meta-analysis [17], was performed for GAS-related variants in the TNF-α gene and OSAHS.

## Materials and Methods

We performed the present meta-analysis according to the guidelines of Preferred Reporting Items for Systematic Reviews and Meta-Analysis (PRISMA).

### Publication search strategy

To identify all eligible studies that address the association between the TNF-α-308 G/A polymorphism and OSAHS, we systematically searched major electronic literature databases, including PubMed, Embase, China National Knowledge Infrastructure (CNKI), Wanfang and Weipu. The last search was updated to March 2013. The following search terms were used: “obstructive sleep apnea hypopnea syndrome” or “OSAHS” or “obstructive sleep apnea syndrome” or “OSAS” or “obstructive sleep apnea” or “OSA” and “tumor necrosis factor” or “TNF” or “tumor necrosis factor-α” or “TNF-α” in combination with “gene” or “polymorphism” or “variants” or “alleles”. Additionally, we manually searched for relevant published studies and review articles. No language restrictions were applied.

### Inclusion and exclusion criteria

Included studies met the following criteria for the present meta-analysis: (1) evaluation of the TNF-α-308 G/A polymorphism and OSAHS susceptibility, (2) case-control or cohort design, and (3) sufficient data to determine the genotype distributions. Studies were excluded for the following reasons: (1) absence of control subjects, (2) non-clinical studies, (3) reviews, abstracts, or conference papers, (4) no reported genotype distribution or allele frequency data and participants were not adults (Age ≤18 years old). We accepted the definition of OSAHS provided by authors in each included study.

### Data extraction

Two investigators (Drs. Zhong and Xiong) independently extracted required data from all the eligible studies. The following data were collected from each included study: first author’s name and publication year, country, ethnicity, genotyping method; age, body mass index (BMI), gender distribution, and apnea-hypopnea index (AHI) of OSAHS and controls; genotype frequency in cases and controls. We also extracted the following additional information: case definition, case and control selection. In addition, it was recorded whether the genotyping in each study was blinded to clinical status. If a discrepancy existed in data collecting, it was resolved by group discussion. If queries or further study details were needed regarding the included studies, the respective authors were contacted via electronic mail.

### Quantitative data analysis

This meta-analysis examined the association between TNF-α variant and the risk of OSAHS on the basis of the allele contrast (mutant type vs. wild type) [17]. The associations were indicated as pooled OR with the corresponding 95% confidence interval (CI). In addition, the generalized odds ratio (ORG) was calculated for each study [18]. The testing of a genetic association using the ORG is powerful even in the absence of Hardy–Weinberg equilibrium (HWE) [19]. In synthesizing the studies, the random-effects (RE) pooled OR of the allele contrast and the RE ORG were used [18]. RE modeling assumes a genuine diversity in the results of various studies. Then, the estimation of pooled OR incorporates the between-study variance. When there is lack of heterogeneity, the RE model coincides with the fixed-effects model [17], however, the RE is preferable for statistical inference more conservative. Thus, in the present meta-analysis, the RE model was used. The heterogeneity between studies was tested using the Q-statistic [20]. If PQ was less than 0.10, then heterogeneity was considered statistically significant. Heterogeneity was quantified with the I^2^ metric, which is independent of the number of studies in the meta-analysis [21]. Sensitivity analysis, which examines the effect of excluding specific studies, was also performed [17]. The distribution of the genotypes in the healthy control group was tested to determine whether it is in Hardy–Weinberg equilibrium (HWE), an indicator of the quality of a study, Studies with the controls not in HWE were subjected to a sensitivity analysis [17]. Subgroup analyses were carried out by ethnicity. Furthermore, the mode of inheritance was estimated using the degree of dominance index (h-index) [22], [23]. The h-index is defined as the ratio of the natural logarithms of the ORs of the two orthogonal contrasts (models): the co-dominant and the additive models. A cumulative and recursive cumulative meta-analysis was carried out for each polymorphism to evaluate the trend of the risk effect (i.e. OR) of the allele contrast in time [17]. In cumulative meta-analysis, studies were chronologically ordered by publication year, and then the risk effect was obtained at the end of each year, that is, at each information step. In recursive cumulative meta-analysis, the relative change in pooled OR in each information step was calculated. All statistical analyses were performed by Stata (ver. 11.0, Stata Corporation, College Station, TX) and ORGGASM software (http://biomath.med.uth.gr) for the meta-analysis. A P value<0.05 was considered statistically significant.

## Results

### Search results

**Figure 1:**
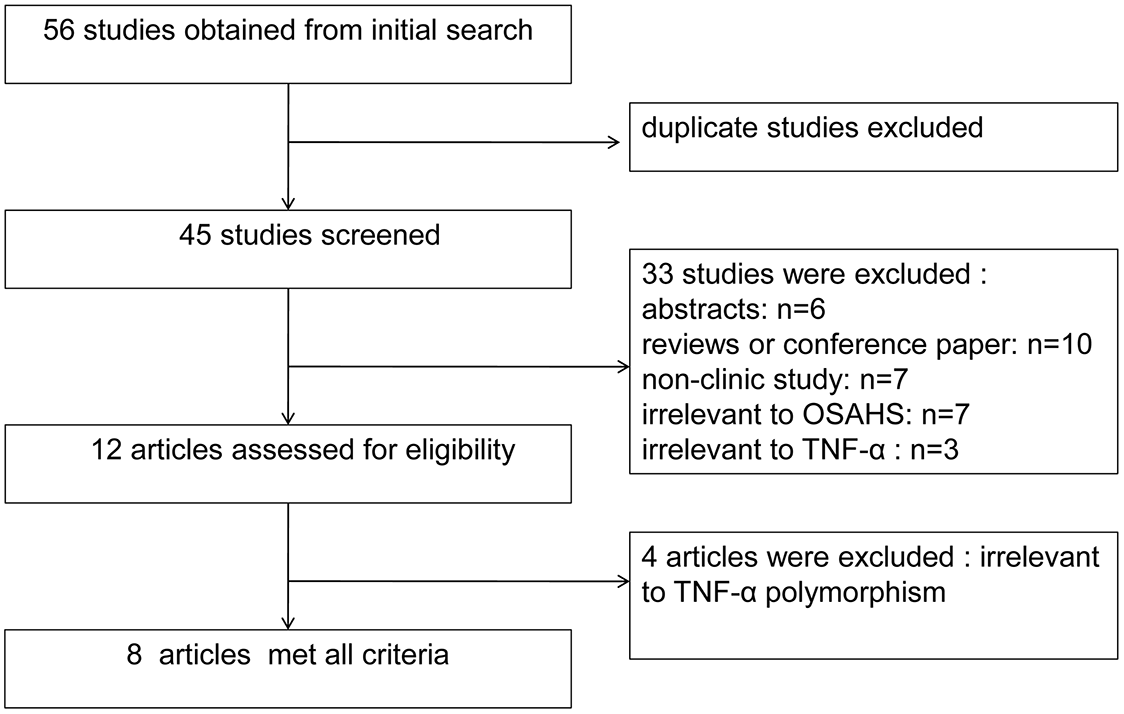
Flow chart of literature search and study selection.

After searching all the electronic databases described above, 56 articles were obtained after the initial search. Forty-five studies were screened after duplicate studies were excluded. Of these, six were abstracts, 10 were review articles or conference paper, and 7 articles were not clinical studies. Additionally, seven of these studies were not relevant to TNF-α, three were not relevant to OSAHS, one study was focused on children, and four were not relevant to the TNF-α polymorphism. Finally, seven articles were included in our meta-analysis (Figure 1).

### Study characteristics

Table 1 and table 2 summarize the characteristics of all of the included studies. The publication year ranged from 2005 to 2013. Half of the articles included were from Asia [11], [12], [13], [14]: three from China [12], [13], [14] and one from India [11]. The other studies were from western countries: one from the United Kingdom [16], one from Poland [15], and one from Greece [10]. Regarding ethnicity, four studies concerned patients of Asian origin [11], [12], [13], [14], three concerned patients of Caucasian origin [10], [15], [16]. For the determination of the genetic polymorphism, validated genotyping methods were used in all studies. The cases and controls of included studies were mainly middle-aged and slightly overweight, although one study did not report BMI in controls [15]. AHI of cases ranged from ≥21 to 57.5±16.3 events/h; the AHI of controls were all below 5 (Table 1). Of the 7 studies, one study was not in HWE [11]. All studies provided unadjusted estimates of risk effect, and therefore, adjusted pooled ORs were not calculated.

**Table 1:** Characteristics of included studies in the meta-analysis.

Author (year)	Country	Ethnicity	Genotyping	OSAHS subjects				Control subjects				p_HWE_
				Age	BMI	Gender(M/F)	AHI(events/h)	Age(year)	BMI(Kg/m^2^)	Gender(M/F)	AHI(events/h)	
Almpanidou(2012)	Greek	Caucasian	DNA sequencing	51.0±12.4	31.4±5.24	198/22	42.6±27.5	50.6±13.8	29.6±4.3	278/41	3.3±2.2	0.11
Bhushan(2009)	India	Asian	PCR-RFLP	46±18	31.5±4	84/20	48±25	44±10	31±4	65/38	2.8±1.7	<0.01
Guan (2013)	China	Asian	PCR-RFLP	43.6±11.7	27.4±3.4	452/79	49.9±26.8	42.6±13.1	26.89±3.7	126/36	<5	0.60
Li(2013)	China	Asian	DNA sequencing	45.0±9.0	29.4±2.1	155/0	57.5±16.3	46.3±8.0	24.1±2.3	100/0	<5	0.52
Liu(2006)	China	Asian	PCR-RFLP	44.3±9.8	26.3±3.5	67/9	≥5	41.7±10.1	25.7±3.3	37/5	<5	0.07
Popko(2008)	Poland	Caucasian	PCR-RFLP	21∼27	>25	74/28	≥5	18∼65	NR	39/38	<5	0.25
Riha(2005)	UK	Caucasian	TaqMan	52±9	30±6	83/20	≥15	51±10	27±5	33/30	NR	0.34

Abbreviation: OSAHS, obstructive sleep apnea-hypopnea syndrome; NR, not reported; BMI, body mass index; AHI, apnea-hypopnea index; HWE, Hardy–Weinberg equilibrium; PCR: polymerase chain reaction; PCR-RFLP: PCR-restriction fragment length polymorphism.

**Table 2:** Genotype distributions in case and control groups.

Study	Case/control	case			control			Outcome	ORG(95% CI)
		GG	GA	AA	GG	GA	AA		
Riha(2005)	103/190	52	44	7	130	52	8	Increased risk	2.01(1.27–3.19)
Liu(2006)	76/42	45	23	8	35	6	1	Increased risk	3.35(1.37–8.23)
Popko(2008)	102/77	73	29	0	59	18	0	Increased risk	1.28(0.66–2.51)
Bushan(2009)	104/103	74	22	8	90	10	3	Increased risk	2.70(1.35–5.42)
									2.38(1.22–4.64)*
Almpanidou(2012)	220/319	118	83	19	221	84	14	Increased risk	1.90(1.35–2.65)
Guan(2013)	531/162	430	95	6	143	18	1	Increased risk	1.76(1.04–2.97)
Li(2013)	155/100	137	18	0	88	12	0	No association	0.95(0.44–2.04)

Note: *mean an adjustment for HWE.

### Main results, subgroup analysis, and sensitivity analyses

**Figure 2:**
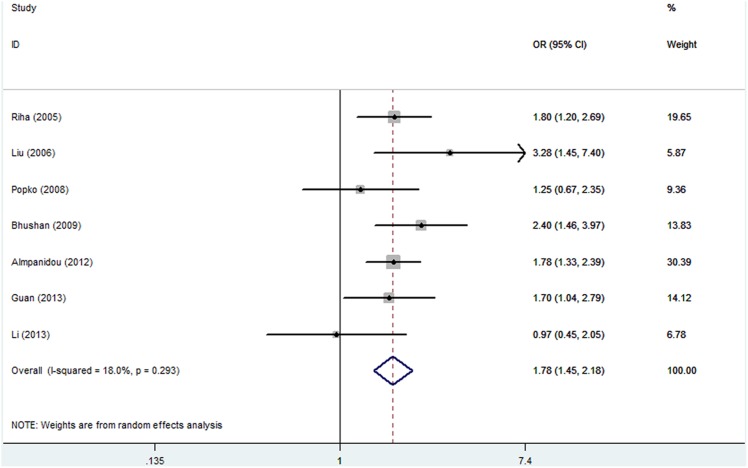
Random-effects (RE) model. Odds ratio (OR) estimates with the corresponding 95% confidence interval (CI) for the allele contrast.

For the allele contrast, there were 7 articles evaluating the association between TNF polymorphism and the risk of OSAHS. The results showed no heterogeneity among studies (I^2^ = 18. 0%, PQ = 0.293), and the random-effects OR (95% CI), 1.78 (1.45–2.18; Figure 2). The sensitivity analysis (exclusion of studies with controls not in HWE (Bushan) also showed no heterogeneity among studies (I^2^ = 13.0%, PQ = 0.332), and the random-effects OR was also significant: OR (95% CI), 1.70(1.37–2.10). An adjustment for HWD was performed, and the result did not change. In subgroup analysis, namely, no heterogeneity existed and significant associations were found in Caucasian OR (95% CI), (1.71, 1.37–2.13). In Asian, heterogeneity existed and significant associations were found (Table 3).

**Table 3:** Comparison of different genetic models for -308G/A polymorphism and OSAHS risk in subgroups.

Subgroup analysis	No of studies	No of cases	No of controls	I^2^	PQ	ORG (95% CI)
All model-free	7	1291	993	15.6	0.31	1.84(1.47–2.32)
All in HWE	6	1187	890	14.6	0.32	1.77(1.40–2.25)
Caucasian	3	425	586	0.0	0.52	1.83(1.42–2.35)
Asian	4	866	407	48.2	0.12	1.92(1.18–3.13)
						1.86(1.18–2.94)*
All allele	7	1291	993	18	0.29	1.78(1.45–2.18)
Caucasian	3	425	586	0.0	0.58	1.71(1.37–2.13)
Asian	4	866	407	48.8	0.12	1.90(1.24–2.93)

Abbreviation: HWE, Hardy–Weinberg equilibrium. Note: *mean an adjustment for HWE.

In the model-free approach, the results showed no heterogeneity among studies (I^2^ = 16%, PQ = 0.31), and the random-effects model was used: ORG (95% CI), 2.01 (1.27–3.19). Similarly, the result of the sensitivity analysis (exclusion of studies with controls not in HWE (BUSHAN) was ORG (95% CI): 1.77 (1.40–2.25), with no heterogeneity (I^2^ = 14.6%, PQ = 0.32). An adjustment for HWD was performed, and the result did not change. In subgroup analysis, significant association was found in both Caucasians [ORG (95% CI):1.83 (1.42–2.35); I^2^ = 0, PQ = 0.52] and Asian, [ORG ORG (95% CI):1.92 (1.18–3.13); I^2^ = 35%] (Table 3).

The additive and codominant models also produced significant ORs (unadjusted and corrected for deviation from HWE). The mode of inheritance, after correcting for deviation from HWE, was domiance of allele A (h = 0.50).

### Potential bias

**Figure 3:**
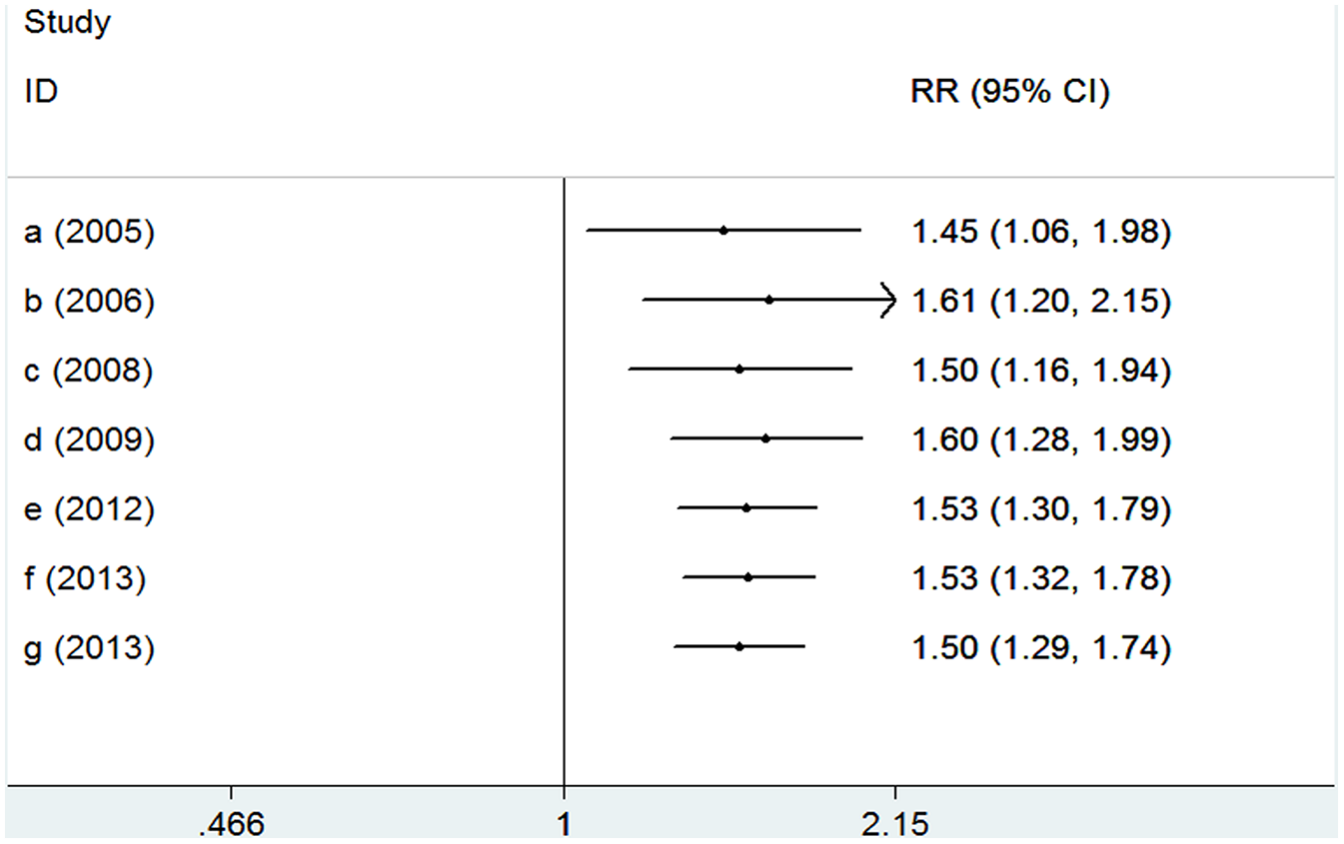
Cumulative meta-analysis for the allele contrast of TNF-α variants and the risk of OSAHS. The pooled odds ratio (OR) with the corresponding 95% confidence interval (CI) at the end of each year-information step is shown.

**Figure 4:**
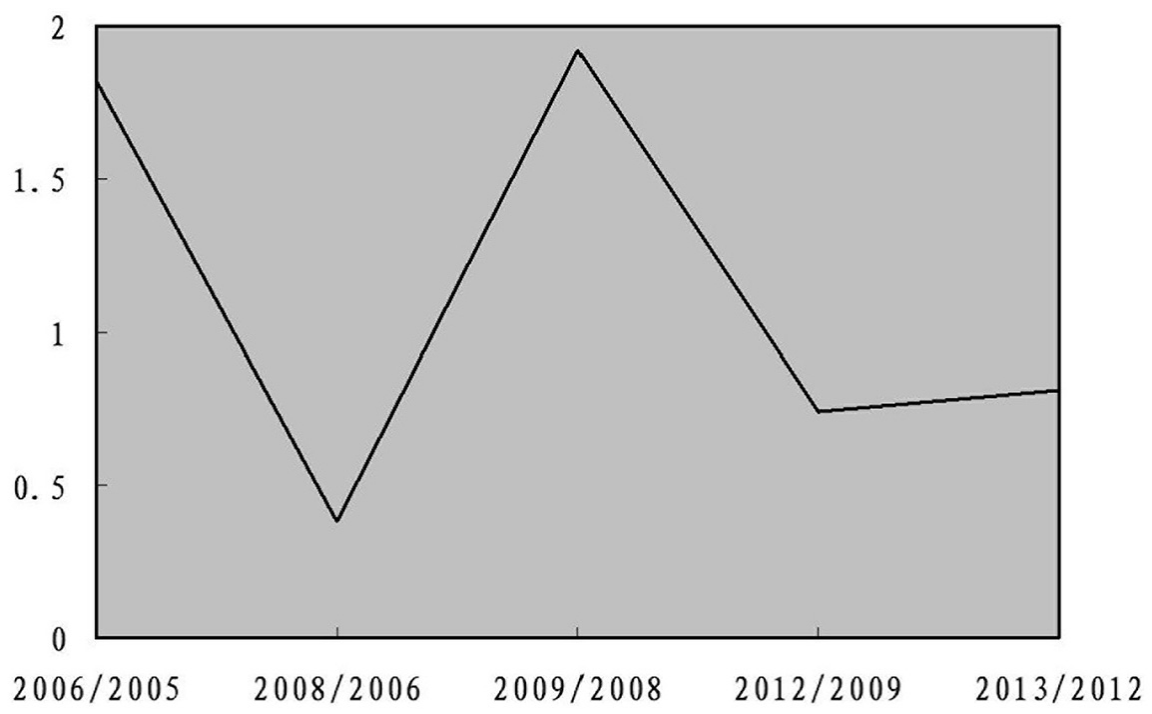
Recursive cumulative meta-analysis for the allele contrast. The relative change in the random effects pooled odds ratio (OR) in each information step (OR in next year/OR in current year) is shown.

None of the studies included in the meta-analysis stated that genotyping was performed blinded to clinical status. In the cumulative meta-analysis, the pooled genetic risk effect remained significant in the entire period studied. However, the recursive cumulative meta-analysis did not show stability in pooled OR change (Figure 3 and Figure 4). Thus, there is a trend of an association and more evidence is needed to draw a safe conclusion on the significance and magnitude of the effect size. Harbold’s test indicated that there was no differential magnitude of effect in large versus small studies for all settings (P>0.10).

## Discussion

This meta-analysis examined TNF-α polymorphism and its relationship to susceptibility for OSAHS. The strength of the present analysis was based on the accumulation of published data giving greater information to detect significant differences. Significant associations were found either for the allele contrast or for the model-free approach. In addition, the consistency of genetic effects across populations from different ethnicities was investigated. Main and subgroup analyses in Caucasian and Asian for the allele contrast and the model-free for polymorphisms have produced significant results. There was consistency of genetic effects across different ethnicities, because they were not differentiated from the overall effect.

Currently, meta-analyses of genetic association studies are usually based on the analysis of various genetic models. However, these models are not independent. In addition, there are no prior biological such as the dominant, recessive, additive, and codominant models; however, these models are not independent satisfactions for the choice of a specific genetic model, and the interpretation of results is complicated when more than two models are significant [23], [24]. But in our study, the model-free approach was used. And it could provide an integrated way to evaluate genetic associations by exploiting all available information in terms of clinical status (case–control) and genotype distribution. ORG is a metric that quantifies the probability of disease given that a disease subject has a higher or lower mutational load than a healthy one. The application of ORG might overcome the drawbacks of multiple model testing or erroneous model specification and make the interpretation of the results easier [18]. The model-free approach (ORG = 2.01) indicated that for any two participants, diseased and control, the probability of being diseased is 2 folder (relative to the probability of being non-diseased) given that the diseased subject has higher mutational load than the healthy one, those homozygous for the A allele to have Subjects who were homozygous for A allele were considered to have the highest mutational load, those homozygous for G allele to have the lowest, and those who were heterozygous to have an intermediate load [19].

In exploring bias, there was a lack of a differential magnitude of effect in large versus small studies. After the presence of an association was demonstrated, the mode of inheritance was tested using the h-index. The presence of significant association, when the additive model is significant and the co-dominant model is not significant, “non-dominance” or “complete additiveness” is assumed and h = 0. When the co-dominant model is significant (irrespectively of the significant level of the additive contrast), “dominance” is assumed and h>0 or h<0, depending on the risk-position of the heterozygote. The cumulative meta-analysis for TNF-α suggested a consistent significance toward an association as evidence from published studies accumulated. The recursive cumulative meta-analysis indicated instability in the relative change in risk effects, and therefore, more evidence is needed to claim or deny the existence of an association between the variants and OSAHS.

We did not identify any published genome-wide association study in OSAHS; all studies considered here in were candidate gene studies which are known to be susceptible to having limited replication validity [25]. The meta-analysis was based on unadjusted risk estimates as data for possible effect modifiers that influence the estimates of associations (e.g. sex, age, clinical manifestations) were not provided by the individual GAS. In addition, sampling variability in the GAS may be a possible confounding factor on the effect of the genetic variants in OSAHS susceptibility. In Bushan’s study, controls did not conform to the HWE, indicating genotyping errors, population stratification, and selection bias in the recruitment of control participants [17]. In population terms, lack of HWE implies existence of migration, selection, mutation, and absence of random mating; however, their impact in candidate-gene studies is limited. An adjustment for deviations from HWE was performed to avoid overestimation of the genetic risk effects [17].

Although significant associations were detected in our study, the sample sizes were small, restricting the power of the study. Nevertheless, findings of our study could be pooled in a future meta-analysis of multiple studies, providing more power to detect significant associations [17], [26]. The multi-factorial etiology of common disorders involving complex epistemic and gene-environment interactions reduces the likelihood that a single type of studies such as gene-candidate association studies could provide conclusive inferences. Our study could not evaluate gene-gene and gene-environment interactions due to the small number of patients identified. These findings need to be studied in a large cohort of OSAHS patients in the future.

In conclusion, through our meta-analysis, we show a strong association between the distribution of genotypes and alleles of the single-nucleotide polymorphism TNF-α-308 G/A and OSAHS susceptibility. However, additional, well-designed studies using modern genetic investigative techniques (GWAS) and larger sample sizes are warranted to consolidate these findings.

## Supporting Information

Checklist S1 PRISMA Checklist for systematic review and meta-analysis.
(DOC)
